# Acting Assistant Surgeons, U. S. Army

**Published:** 1900-01

**Authors:** 


### ACTING ASSISTANT SURGEONS, U. S. ARMY.

THE Association of Acting Assistant Surgeons, U. S. A., was organised at a meeting held in New York on December 5, 1899. The main object of the association is to secure from congress legislation which will give acting assistant surgeons all the benefits they were promised when contracts were made.

The Treasury Department has ruled that they are entitled to $150 per month and no more, thus cutting out the extra pay received by volunteer officers and enlisted men. When sick and on leave they have not received pay. All agreements made by Surgeon-General Sternberg were thus practically annulled by the Treasury Department decisions. Dr. Sternberg has done and is doing all in his power to regulate this, and has drawn up a bill to give volunteer surgeons the pay due them according to their rank, and are regulating the status of acting assistant surgeons.

The meeting was well attended, harmonious, and the following officers were elected: President, Azel Ames; vice-presidents, Timothy Leary, A. R. Jarrett, T. B. Buffom; secretary, J. P. Warbasse, of Brooklyn; treasurer, Charles W. Brewer; historian, W. S. Terriberry. A legislative committee was also chosen.

---

THE new ordinance recently adopted by the Common Council of Buffalo, and which has so disturbed the faith healers, reads as follows:

> Any person professionally or otherwise in attendance upon or in charge of any person suffering from any infectious or contagious disease, in case a regularly qualified physician has not been called to attend, shall immediately report the existence of such disease and the name and address of the patient or person suffering therefrom to the Board of Health. This section is intended to include any person in whose house or home such patient or sick person may reside.

Surely every good citizen will willingly comply with such a reasonable measure, which is projected and enacted in the interest of the people.

A MEMORIAL tablet to Dr. George H. Rohé is to be erected by the memorial committee of the Maryland Public Health Association. It will be a bronze portrait tablet to commemorate the life and public services of this distinguished physician. It will be placed either in the hall of the Medical and Chirurgical Faculty of Maryland, or at the Springfield Asylum for the Insane, an institution which was erected and organised under the supervision of Dr. Rohé.

---

THE Medical Council of the State of Pennsylvania decided on May 24th, that the practice of osteopathy within the state is illegal, and that those therein engaged are amenable to the law.—*Columbus Med. Jour.*

---

THE annual report of the board of managers of the Craig Colony, for the care of epileptics, located at Sonyea, N. Y., has been submitted to the State Board of Charities.

From statistics it is learned that since the opening of the colony on February 1, 1896, 504 epileptics have been received, of which 378 remain. When the new buildings, now in the course of construction, are completed the capacity of the colony will be increased to a total of 720 beds. These will be filled as soon as they are ready and there will still remain some hundreds of epileptics in almshouses, hospitals and outdoor departments of dispensaries throughout the state.

During the last year 95 new cases were admitted to the institution, 40 men and 55 women. Thirty-nine were discharged, leaving 378 in the institution on October 1st, a total gain of 56 during the year. The death-rate for the last year has been very low, less than 2 per cent., the lowest in the history of the colony. Four insane persons were transferred to state hospitals.

---

THE Supreme Court of the State of Iowa, in a recent decision, holds that the State Board of Medical Examiners has the right to revoke the certificate of a physician if it thinks him incompetent to practise.

---

THE complete suppression of typhoid fever in Berlin, may be regarded as a remarkable sanitary achievement. That it has been accomplished through the perfect filtration of the city's water supply, is a commentary on the imperfections of the systems and sources of water supplied to many American cities. It is to be hoped that Buffalo will put itself abreast of Berlin in this matter without unnecessary delay.

The state care of consumptives was considered at a meeting of a special committee of the State Board of Charities, held at Rochester, December 2, 1899. The members of the committee present were Dr. E. V. Stoddard, of Rochester, and Mr. Harvey W. Putnam, of Buffalo.

The committee was addressed by Drs. John H. Pryor, Henry R. Hopkins and William H. Heath, of Buffalo; Dr. A. M. Veeder, of Lyons; Dr. G. W. Goler, of Rochester, and Messrs. Frederic Almy and Lafayette Long, of Buffalo, all favoring a well matured and effective plan of state care. It is hoped that the committee will report favorably upon the subject.

---

The medical profession and the public in general are to be congratulated upon another conviction obtained in Buffalo for the illegal practice of medicine. It appears that Ferdinand Szymanski was tried recently before Justice Kenefick on the charge of illegally practising medicine. The complaint upon which he was indicted was made last autumn by Dr. J. B. Coakley, chairman of the board of censors of the Medical Society of the County of Erie. The indictment alleged that Szymanski, not being a licensed physician in this country, had administered medicine to and prescribed for Charles S. McDowell, of No. 805 Fillmore Avenue and others.

District-Attorney Penny presented three witnesses. McDowell testified that Szymanski had given him powders for stomach trouble. James Hopkins, who was with McDowell at the time, corroborated that testimony. William Chase testified that Szymanski had diagnosticated his case as one of rheumatism and had given him powders and a prescription on which to obtain other medicine.

Szymanski, in his own defense, declared that he was a licensed physician in the old country. The jury brought in a verdict, finding him guilty as charged in the indictment, but recommending leniency. Justice Kenefick suspended sentence on account of the age and poverty of the defendant.

---

The need of more medical officers in the army has prompted Surgeon-General Sternberg to prepare a bill for presentation to congress providing for the addition to the corps of four assistant surgeon generals with the rank of colonel, ten deputy surgeon generals, with the rank of lieutenant colonel, thirty surgeons with the rank of major and eighty assistant surgeons with the rank of first lieutenant, who shall have the rank of captain after five years of service. It is provided that the vacancies thus created in the grade of colonel, lieutenant colonel and major shall be filled by senority promotion in accordance with established laws and regulations. Acting assistant surgeons to the number authorised are to be appointed, subject to the usual examination, for a probationary period of six months, during which they will attend the Army Medical School in Washington. At the expiration of this time, if their standing is good, they are to be commissioned to fill existing vacancies.

This probationary service is waved in the case of candidates who have rendered satisfactory service as acting assistant surgeons or as commissioned medical officers in the Volunteer Army for six months or more, who may be commissioned at once on passing a satisfactory examination as to their physical, moral and professional qualifications.

---

EYESIGHT in childhood is a subject that deserves the closest attention by parents, guardians and physicians. The *New York Tribune* in a recent issue has printed the following:

An eminent German specialist in this line, Dr. Cohn, has found that the number of nearsighted or myopic pupils shows an increase in every school from grade to grade, and this he attributes to improperly adjusted desks. This progressive increase, according to Dr. Rogers, of Buffalo, N. Y., a close investigator of the subject, is the case in the United States also, and he especially condemns the carelessness with which parents allow their children to begin study without any test of their vision, so that a slight defect is often allowed by neglect to grow into a serious one. In commenting upon this Dr. Rogers lays down the rule that the eyes of all children should be tested before admission as pupils, and if the vision proves to be much below the normal, admission should not be granted until professional counsel has been sought and a certificate meeting the case presented. The same rule, too, should apply to children with inflamed eyes, who should not be admitted until a physician's certificate of the noninfectious nature of the disease is presented.

